# A Hand-book of Materia Medica and Homœopathic Therapeutics

**Published:** 1889-08

**Authors:** 


### A Hand-book of Materia Medica and Homœopathic Therapeutics, by Timothy F. Allen, A. M., M. D., LL.D., etc. Pages 1,165 ; price, $15. Hahnemann Publishing House : Philadelphia, 1889.

Ever since the days of Jahr, attempts have been repeatedly made to *condense* or *abbreviate* the homœopathic materia medica. Most of these attempts have resulted in failure; few of such condensed works have proven useful. They seemed to omit what one needed, and to give just the symptoms which were of no value in prescribing. Dr. Allen's condensation of his *Encyclopædia* seems to be something better than this; as far as a cursory examination can show, it seems to be a useful work. But its true value can only be estimated after a continued use of it in prescribing. The condensation has been very cleverly done; few useful or reliable symptoms seem to be omitted. Some symptoms are rendered a little confused by the method of condensing. But, on the whole, we believe the verdict of students of the materia medica will be in favor of the work. It seems to us Dr. Allen made a great mistake in omitting the "Lacs," Hydrophobinum, etc. He evidently appreciates this, for he offers (see *preface*) to insert them in a new edition, if called for by the profession. Did Dr. Allen omit these remedies for fear of ridicule from the "scientists"?

We have hitherto condemned these *condensed* works, believing them to be the cause of much error in practice. If Dr. Allen's work shall prove so useful as to cause us to make an exception in its favor, we shall be glad. The work, even after a brief study, seems to promise to be useful, therefore we believe few will regret its purchase. The publisher has done his part in good taste, and has given us good paper and type, with strong binding.

